# Bacteria–Host Interactions in Multiple Sclerosis

**DOI:** 10.3389/fmicb.2018.02966

**Published:** 2018-12-04

**Authors:** Davide Cossu, Kazumasa Yokoyama, Nobutaka Hattori

**Affiliations:** ^1^Department of Neurology, Juntendo University, Tokyo, Japan; ^2^Advanced Research Institute for Health Science, Juntendo University, Tokyo, Japan

**Keywords:** bacteria, multiple sclerosis, pathogen–host interaction, innate immunity, acquired immunity

## Abstract

Multiple sclerosis (MS) is caused by a complex interaction of genetic and environmental factors. Numerous causative factors have been identified that play a role in MS, including exposure to bacteria. Mycobacteria, *Chlamydia pneumoniae, Helicobacter pylori*, and other bacteria have been proposed as risk factors for MS with different mechanisms of action. Conversely, some pathogens may have a protective effect on its etiology. In terms of acquired immunity, molecular mimicry has been hypothesized as the mechanism by which bacterial structures such as DNA, the cell wall, and intracytoplasmic components can activate autoreactive T cells or produce autoantibodies in certain host genetic backgrounds of susceptible individuals. In innate immunity, Toll-like receptors play an essential role in combating invading bacteria, and their activation leads to the release of cytokines or chemokines that mediate effective adaptive immune responses. These receptors may also be involved in central nervous system autoimmunity, and their contribution depends on the infection site and on the pathogen. We have reviewed the current knowledge of the influence of bacteria on MS development, emphasizing the potential mechanisms of action by which bacteria affect MS initiation and/or progression.

## Introduction

Multiple sclerosis (MS) has a complex pathophysiology that results from multiple and unclear interactions between genetic and environmental factors. Accumulating evidence over the years also supports the role of infectious agents, particularly viruses, in the etiopathogenesis of MS. Among these, Epstein–Barr virus (EBV) seems to be the strongest candidate as a risk factor (Olsson et al., 2017). In fact, MS risk is higher among individuals with history of mononucleosis or who experienced an EBV infection in childhood (Ascherio and Munger, 2010). Subclinical EBV infection is present in over 95% of all individuals, including those with MS; however, the MS incidence and prevalence differ in each country. Therefore, coinfection with another pathogen, including bacteria, could explain the difference between high- and low-risk areas for MS.

There are several examples of virus–bacteria interactions (Almand et al., 2017), which mainly occur when the pathogens colonize the same site during infection, although sometimes viruses can act at different locations within the host (Steed and Stappenbeck, 2014). For instance, the human immunodeficiency virus targets a wide variety of immune cells, causing depletion of CD4+ T cells and up-regulation of CD14+ cells, which likely contributes to the susceptibility of co-infection with Mycobacterium tuberculosis (Pawlowski et al., 2012). Moreover, during co-infection, the cell wall lipids of mycobacteria can modulate the host cell response, resulting in increased viral propagation and overall worsening of the disease (Pawlowski et al., 2012).

Another kind of virus–bacteria interaction is immune system subversion, such as occurs with the interaction between EBV and periodontopathic bacteria. The latter could induce EBV reactivation via chromatin modification, and virally-infected B cells cause tissue inflammation, decreasing the ability to defend against bacteria, thereby facilitating the progression of periodontal diseases (Gao et al., 2017). A case-control study provided evidence for an association between chronic periodontitis and female patients with MS in Taiwan (Sheu and Lin, 2013). The antigenic epitopes shared between Mycobacterium avium sp. paratuberculosis and EBV, which are cross-recognized by antibodies targeting self-epitopes in MS patients, provided additional evidence of a possible synergistic effect of viral–bacterial coinfection in inducing the pathology (Mameli et al., 2014).

Considering that bacteria are found in many sites of the human body including the brain (which is usually microbe free) and that viruses may efficiently spread from the site of primary infection to other tissues, we speculate that synergistic interactions between multiple pathogens may play a role in the pathogenesis of neuroinflammatory diseases like MS.

There are several possible explanations regarding the role of bacteria or viruses in predisposing an individual to MS autoimmunity. According to the hygiene hypothesis, exposure to pathogenic organisms early in life can confer protective immunity, whereas infections in adulthood can trigger an autoimmune reaction in susceptible individuals (Fleming and Fabry, 2007). Another alternative theory suggests that MS could be caused by a pathogen that is more common in regions of high MS prevalence, where the pathogen is endemic and, in most individuals, causes an asymptomatic infection, but reactivation of the infection several years later can lead to central nervous system (CNS) disease such as MS (Kurtzke and Heltberg, 2001).

In this review, we will describe the current understanding of bacterial pathogens associated with MS and the diversity of mechanisms used by such pathogens to colonize and influence the human CNS.

## Microbe–Host Interactions in MS

Bacteria express specific pathogen-associated molecular patterns (PAMPs), which are recognized by cells of the innate immune system equipped with pattern-recognition receptors (PRRs) (Akira et al., 2006), leading to activation of antigen-presenting cells (APCs), which in turn initiate and direct immune responses against the invading pathogens (Akira et al., 2006). Activated APCs that contain self-antigens obtained from dying cells or damaged tissue, might activate autoreactive T and B cells. PRRs are expressed either on the cell surface or intracellularly and include Toll-like receptors (TLRs), nucleotide-binding oligomerization domain (NOD)-like receptors (NLRs), C-type lectin receptors (CLRs), and retinoic acid-inducible gene-I (RIG-I)-like receptors that primarily sense viral RNA.

Toll-like receptors are transmembrane proteins that are located in both the cell membrane (TLR1, TLR2, TLR4, TLR5, TLR6, and TLR10) and in endosomal vesicles (TLR3, TLR7, TLR8, and TLR9) of immune cells, such as macrophages and dendritic cells (Bsibsi et al., 2002). TLRs detect different microbial components, and their activation initiates intracellular signaling cascades that trigger inflammatory mediators. TLRs are expressed in both resident CNS glia and infiltrating immune cells, and an increase in TLR2 expression has been observed in MS demyelinating brain lesions, where the TLR1/TLR2 and TLR6/TLR2 heterodimers recognize tri- or diacyl lipopeptides from bacteria, respectively (Sloane et al., 2010). In experimental autoimmune encephalomyelitis (EAE), which is an animal model of MS, TLR signaling through the myeloid differentiation primary response protein 88 (MyD88)-dependent pathway plays a significant role in the development of the disease (Miranda-Hernandez and Baxter, 2013). Human MyD88 is the canonical adaptor protein for inflammatory signals used by all TLRs (except TLR 3) (Miranda-Hernandez and Baxter, 2013). Helminth antigens modulate immune responses in B cells and dendritic cells isolated from parasite-infected MS patients via TLR2, through distinct signaling pathways including the MyD88-dependent pathway (Correale and Farez, 2012). Cell-surface human TLR2 recognizes a broad range of bacterial PAMPs comprising heat shock proteins and peptidoglycans; hence, TLR2 is likely related to MS pathogenesis. TLR9 (an endosomal receptor that senses CpG DNA from bacteria and viruses) and MyD88 are essential modulators of the autoimmune process during the effector phase of EAE. Human TLR9 seems to play both protective and harmful roles at different stage of MS (Zhou et al., 2017).

Nod-like receptors (NLRs) are intracellular proteins that bind peptidoglycans present in the bacterial cell wall (Gharagozloo et al., 2018). Nucleotide binding oligomerization domain (NOD)1 detects gram-negative bacteria such as *Chlamydia* or *Helicobacter pylori*, whereas NOD2 is involved in recognizing mycobacteria (Gharagozloo et al., 2018). NLR family members are positive and negative regulators of inflammatory responses, and mutations in the NLRP1 gene have been linked to inflammatory disorders including MS (Maver et al., 2017).

C-type lectin receptors (CLRs) are expressed on the surface of APCs and bind carbohydrates on pathogen surfaces. Mice deficient for dendritic cell immunoreceptor (DCIR), one CLR implicated in the suppression of T cell function, showed an exacerbation of EAE, suggesting that CLR regulation is important for the development of autoimmune diseases such as MS (Seno et al., 2015).

Pathogenic bacteria have evolved various mechanisms to colonize the host, which influence their impact on MS. Bacteria can adhere and multiply at the surfaces of epithelial cells due to the hydrophobicity of the cell wall or the presence of specific surface molecules (Ribet and Cossart, 2015).

**FIGURE 1:**
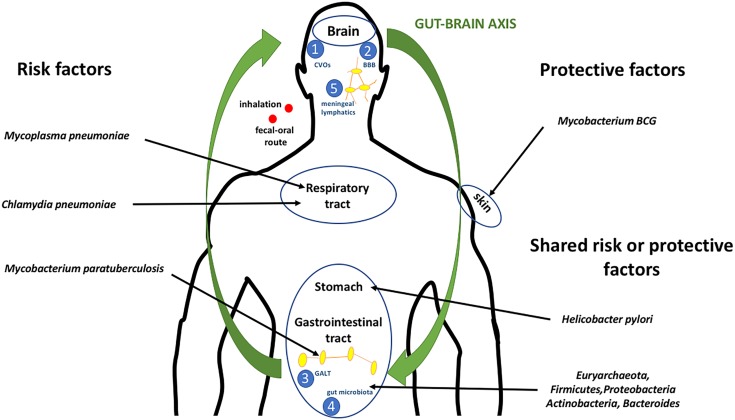
Microbe–host interactions in MS. Bacteria gain access to the body by penetrating the mucous membranes of the respiratory and/or gastrointestinal tracts, or by direct inoculation. Microbial antigens can cross-activate peripheral T cells through different mechanisms, such as molecular mimicry, bystander activation, or epitope spreading. Naïve T cells recognize myelin and rearranged TCRs, and certain myelin-reactive CD4+ T cells can differentiate into Th1 and Th17 cells, then gain access to the CNS, where they are reactivated by APCs and have harmful effect on the CNS parenchyma. Furthermore, intestinal dysbiosis causes an imbalance between beneficial and pathogenic enteric bacteria, contributing to immune dysregulation in the periphery and subsequently influencing CNS immune tolerance. Bacteria may communicate with the brain through various routes, including: 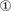 through circumventricular organs characterized into sensory and secretory organs, comprising the subfornical organ, vascular organ of the lamina terminalis, area postrema, median eminence, neurohypophysis, sub-commissural organ, choroid plexus, and pineal gland (Weiss and Schaible, 2015); 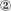 through the BBB or the blood–cerebrospinal fluid barrier (Ganong, 2000); 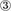 through the GALT, which consists of Peyer’s patches, intraepithelial lymphocytes, and lamina propria lymphocytes (Doran et al., 2013); 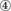 through gut microbiota by a bi-directional communication system (gut–brain axis), including the autonomic nervous system, the enteric nervous system, the vagus nerve, and the hypothalamic pituitary adrenal axis (Wucherpfennig, 2001); and 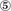 through meningeal lymphatics capable of draining CNS macromolecules into the cervical lymph nodes. Inflammation is known to induce expansion of the local lymphatic vasculature in peripheral tissues and, hence, it is likely that bacterial exposure autoimmune cell activation will occur (Cosorich et al., 2017).

Intracellular bacteria can enter and proliferate inside host cells, where they can neutralize the humoral and complement-mediated immunity (Weiss and Schaible, 2015). Mycobacteria, especially, have developed different strategies to maintain intracellular infection, blocking the acidification of the phagosome and its fusion to lysosomes (Weiss and Schaible, 2015). Bacteria may cross epithelial or endothelial host barriers and gain access to internal tissues, except for the CNS (Figure 1).

Microglia are resident macrophages of the CNS that represent the first line of defense in response to pathogens. Circumventricular organs are structures that permit substances such as hormones to leave the brain without disrupting the blood–brain barrier (BBB) and allow microglia to quickly sense signs of infection through TLRs, NLRs, and scavenger receptors (Ganong, 2000). It has been demonstrated that some pathogens including *Mycobacterium tuberculosis*, *Streptococcus pneumoniae*, and others can penetrate the BBB or the blood–cerebrospinal fluid barrier and enter into the CNS, altering its permeability by releasing toxic cell wall components (Dando et al., 2014). This process promotes further production of inflammatory factors by activated microglia, which have either neurotoxic or neuroprotective functions, depending on the stage of the disease (Doran et al., 2013).

The intestinal epithelium constitutes a target for several pathogens. Gut-associated lymphoid tissue (GALT) functions in the immune system to protect the body from bacterial invasion in the gut. GALT includes Peyer’s patches containing antigen-sampling microfold (M) cells, which are necessary for the initiation of antigen-specific immune responses and represent a route of entry to deeper tissues used by different pathogens (Wucherpfennig, 2001).

Mounting evidence also indicates that the microbiota has an important impact in bidirectional interactions between the enteric nervous system and the CNS. Some bacteria like commensal *Clostridia* are strong inducers of T regulatory cells and maintaining gut homeostasis, whereas other bacteria contribute to Th17 cell expansion in patients with MS (Cosorich et al., 2017).

Interactions between pathogens and the innate immune system could take place in the meningeal lymphatics, a vascular system that enables the passage of macromolecules and brain-derived immune cells into the cervical lymph nodes (Louveau et al., 2015). Lymph nodes are organs of the lymphatic system where APCs present bacterial antigens to lymphocytes. Since these vessels represent an important pathway for APCs from the brain to lymph nodes, theoretically self-reactive T cells can be primed against brain antigens and therefore result in an autoimmune attack.

## Mechanisms of Autoimmune Disease Induction

Host–pathogen interactions can have different consequences depending on several factors such as host genetic background, mucosal barrier dysfunction, bacterial virulence, and transmissibility. Bacterial infections can lead to significant activation of APCs, which can promote the expansion of pre-primed autoreactive T and B cells, initiating the autoimmune process. The inflammatory process may also occur in the absence of persistent infections by an immune response directed against post-infection antigens present in the target tissues. These antigenic components might cross-react with self-antigens, thereby altering the regulatory state of the host, despite the pathogen already being cleared by the natural immune responses. Several mechanisms based on bacterial antigens have been proposed to explain how pathogens might trigger autoreactive immune responses in MS.

The molecular mimicry theory states that foreign peptides with sequence similarity to self-peptides can activate pathogen-derived autoreactive T cells (Oldstone, 2014). This process is mediated by T cell receptors (TCRs) on the surface of T cells or natural killer T (NKT) cells, which recognize bacterial peptides fragments bound to major histocompatibility complex (MHC) molecules on the surface of APCs or lipids/glycolipids presented by CD1 molecules (Oldstone, 2014).

Invariant NKT (iNKT) cells are involved in the molecular recognition of microbial lipid-based antigens complexed with CD1d, such as the *Borrelia burgdorferi* glycolipid antigen BbGL-2 (Podbielska et al., 2018), which is structurally similar to galactocerebroside, the major component of myelin. iNKT cells play an important role in linking innate and adaptive immune responses, and various reports have demonstrated functional defects in iNKT cells in MS (Podbielska et al., 2018).

Another example of the cross-reactivity phenomenon is that T cell clones from MS patients recognize the immunodominant myelin basic protein (MBP)_89–101_ peptide bound to human leukocyte antigen (HLA)-DR2, which is associated with MS susceptibility (Li et al., 2005). Different studies have shown that microbial peptides can induce MS-like disease in rodents through mechanisms of molecular mimicry in EAE (Oldstone, 2014).

Interestingly, in EAE, inoculation of the encephalitogenic antigen requires co-administration of an adjuvant to increase its immunogenicity and efficacy. Complete Freund’s adjuvant (CFA) containing heat-killed *Mycobacterium tuberculosis* suspended in an oil base activates APCs through TLR2 due to mycobacterial components that induce a strong Th1 cell response, which results in augmented delayed-type hypersensitivity against autoantigens (Billiau and Matthys, 2001).

*Bordetella pertussis* toxin is a key substance necessary for the induction of EAE in mice after active immunization, as it can modulate the permeability of the BBB and thus facilitate pathogenic T cell migration into the CNS. Pertussis toxin specifically stimulates TLR4 and the inflammasome (Hofstetter et al., 2002). Of note, this microbial toxin can contribute to the initiation of EAE by promoting antigen recognition by pathogenic T cells on activated microglia in the CNS, without a molecular mimicry mechanism (Hofstetter et al., 2002). For example, injecting pertussis toxin in myelin-specific TCR transgenic mice was sufficient to induce EAE without CFA (Bettelli et al., 2003).

An additional process of enhanced autoantigen presentation during infection is epitope spreading, in which T cell responses against a single antigenic peptide can vary during an inflammation by priming T cells specific for other self-peptides (Floreani et al., 2016). MS progression is influenced by intermolecular and intramolecular T and B cell epitope spreading. Different autoantigen epitopes may be important for each MS patient, depending of his or her MHC background, and pathogenic autoantibodies could be generated by cross-reactivity against the tertiary structure of microbial antigens. This process can cause tissue damage and the exposure to self-antigens or new epitopes, which are usually hidden, leading to a secondary autoimmune response with the production of antibodies against newly released antigens.

Another interactive mechanism by which bacteria can influence the host immune system is through the engagement of super antigens. Bacterial super antigens can initiate bystander activation, stimulating T cells specific for self-antigens without any requirement for antigen processing (Kreiss et al., 2004). Since T cell activation by a super antigen is dependent only on specific TCR V beta elements in association with MHC class II molecules, distinct super antigens can activate more T cells than immunodominant peptides. Mycobacteria, mycoplasma, and enteric microbiota-generated super antigens such as staphylococcal enterotoxin B (SEB), some of which are involved in EAE disease exacerbation (Kreiss et al., 2004). While the role of SEB in the etiology of MS is currently unknown, it has been implicated in the reactivation of EAE. An example is the injection of mice with SEB, which causes a clinical relapse in MBP-immunized mice that had already recovered from a previous episode of EAE (Brocke et al., 1993). In contrast, the treatment of PL/J mice with SEB protected PL/J mice from the development of EAE (Soos et al., 1993).

## Role of Bacteria in MS: Good Guys or Bad Guys?

Since the list of pathogens associated with MS has grown significantly over the past 20 years, we have provided an updated list of the bacteria and their suspected role in the disease pathogenesis (Table 1), focus on bridging innate and adaptive immunity.

**Table 1:** Summary of the major bacteria linked to MS.

	Bacteria	Target TLR	Relationship with EAE	Relationship with MS	Suspected mechanism of action
Risk factor	*Mycobacterium avium* subsp. *paratuberculosis*	TLR2 (Arsenault et al., 2014; Cossu et al., 2017a) TLR9 (Arsenault et al., 2013; Cossu et al., 2017a)		DNA and antibodies in serum (Cossu et al., 2011, 2014, 2015, 2017b), intrathecal IgG synthesis in CSF (Mameli et al., 2016)	Activation of autoreactive T cells in the periphery by and reactivation in brain (Cossu et al., 2013, 2017a)
	*Chlamydia pneumoniae*	TLR2 (Shimada et al., 2012) TLR3 (Shimada et al., 2012) TLR4 (Shimada et al., 2012) TLR9 (Shimada et al., 2012)	Exacerbation of disease severity (Du et al., 2002)	DNA and antibodies in CSF (Contini et al., 2008; Fainardi et al., 2008; Tang et al., 2009; Ivanova et al., 2015)	Infection of neuronal glial cells and alteration of BBB permeability (MacIntyre et al., 2002)
	*Mycoplasma pneumoniae*	TLR1 (Waites and Talkington, 2004) TLR2 (Waites and Talkington, 2004) TLR6 (Enevold et al., 2010)		Antibodies in serum, intrathecal IgG synthesis in CSF (Bahar et al., 2012) Isolation from brain and urine samples of MS patients (Harbo et al., 2013)	Direct invasion in the brain may cause demyelination (Lindsey and Patel, 2008)
	*Clostridium perfringens* type B	TLR2 (Abramovitz et al., 1987)		Immunoreactivity to epsilon toxin in PBMCs and CSF (Dorca-Arévalo et al., 2008) Bacterium isolated from feces of MS patients (Dorca-Arévalo et al., 2008)	The neurotoxin affects endothelial cells, myelinated fibers, and oligodendrocytes of the CNS (Sakai et al., 2011)
	*Helicobacter pylori*	TLR4 (Efthymiou et al., 2017)		Histological presence of infection (Gavalas et al., 2015) Serum antibodies (Efthymiou et al., 2017)	Release of pro-inflammatory mediators and loss of self-tolerance (Kountouras et al., 2015)
	*Euryarchaeota, Firmicutes*, *Proteobacteria (Sutterella)*	TLR2 (Schmutzhard et al., 1988) TLR4 (Schmutzhard et al., 1988) TLR5 (Schmutzhard et al., 1988) TLR9 (Schmutzhard et al., 1988)	Exacerbation of disease severity (Carabotti et al., 2015) Increased disease incidence in a spontaneous mouse model (Shahi et al., 2017)	More frequent in MS patient’s microbiota than healthy controls (Colpitts and Kasper, 2017; Shahi et al., 2017; Tremlett and Waubant, 2017)	Pro-inflammatory response and reduced IL10 production (Carabotti et al., 2015) Staphylococcal enterotoxin B (Brocke et al., 1993)
Protective factor	*Mycobacterium bovis BCG*	TLR2 (Cossu et al., 2017a) TLR4 (Cossu et al., 2017a)	Suppression of disease (Sewell et al., 2003; Lee et al., 2008)	Reduced MRI activity and lower antibody titer in MS patients, compared to control subjects (Cossu et al., 2014, 2017b; Ristori et al., 2014)	Skin vaccination or direct inoculation in brain or gut of mice (Sewell et al., 2003; Lee et al., 2008; Moliva et al., 2017) Antigenic competition, diversion of autoreactive T cells to granulomas (Sewell et al., 2003)
	*Helicobacter pylori*	TLR4 (Efthymiou et al., 2017)	Infection reduces disease severity (Cook et al., 2015)	Lower antibody titer in MS patients compare to controls (Yoshimura et al., 2013; Jaruvongvanich et al., 2016; Yao et al., 2016)	Suppression of Th1/Th17 cell responses (Salama et al., 2013)
	*Firmicutes (Clostridia), Actinobacteria, Bacteroides*	TLR2 (Schmutzhard et al., 1988) TLR4 (Schmutzhard et al., 1988) TLR5 (Schmutzhard et al., 1988) TLR9 (Schmutzhard et al., 1988)	Reduced disease susceptibility following oral administration of *Bacteroides fragilis* PSA (Carabotti et al., 2015)	Less frequent in the microbiota of MS patients than in healthy control subjects (Logsdon et al., 2018)	Anti-inflammatory response (Carabotti et al., 2015) Expansion of CD39(+) Tregs and increased IL10 expression (Carabotti et al., 2015)

## Mycobacteria

Various intracellular mycobacteria of the same genus have been associated with MS, but different species seem to play different roles in the disease pathology.

*Mycobacterium avium* subsp. *paratuberculosis* (MAP) is a non-tuberculous mycobacterium that causes paratuberculosis in ruminants and has been proposed as the causative agent of Crohn’s disease in humans; however, growing evidence has implicated it as a candidate trigger of MS (Cossu et al., 2017a).

Human studies conducted in Italy and Japan have shown a higher antibody titer against specific proteins and peptides in the sera and cerebrospinal fluid (CSF) of patients with MS, compared to patients with other diseases and healthy control groups (Cossu et al., 2013, 2017a). Bacterial peptides could also ignite a T cell response in peripheral blood mononuclear cells (PBMCs) isolated from relapsing–remitting MS subjects (Cossu et al., 2015). In addition, intrathecally synthesized IgGs reactive to these MAP-derived peptides were detectable in MS patients (Mameli et al., 2016). Bacterial DNA was isolated from MS patients by nested PCR (Cossu et al., 2011), but the bacteria were never isolated and cultured from the MS subjects. MAP colonizes the gastrointestinal tract, and MAP exposure from cattle to humans primarily occurs via the fecal–oral route through contaminated milk and dairy products (Cossu et al., 2017a). During an infection or antigen exposure, autoreactive T cells activated in the periphery by molecular mimicry might penetrate the BBB and be reactivated in the CNS by local APCs (Cossu et al., 2013).

*Mycobacterium avium* subsp. *paratuberculosis* invasion appears to occur mainly through M cells of the Peyer’s patches, after which it invades intra-epithelial macrophages where it can expand. TLR2 and NOD2 have been implicated in the recognition of mycobacteria through the binding of mannosylated lipoarabinomannans and peptidoglycans, respectively (Arsenault et al., 2014). Moreover, MAP can inhibit TLR9 and MyD88 signaling, and alter the expression of IFNγ receptors to overcome both innate and acquired immune responses in cattle (Arsenault et al., 2013). The roles of different TLRs and MyD88-mediated immune responses in MS and the effect of MAP antigens on EAE are still under investigation. It has been hypothesized that chronic infection with MAP in the human gastrointestinal tract in some individuals can cause the spreading of proinflammatory mediators, which, in turn are responsible for initiating inflammation in the brain and driving polarization of the immune response toward a Th1/Th17 phenotype (Cossu et al., 2013).

*Bacillus Calmette–Guérin* (BCG) is a live attenuated strain of *Mycobacterium bovis*, the etiological agent of tuberculosis in animals and occasionally in humans (Cossu et al., 2017a). The main use of BCG is for vaccination against tuberculosis, and many groups have investigated the role of the bacteria in preventing or reversing autoimmune diseases such as MS.

Generally, the magnitudes of humoral responses elicited by BCG antigens in MS patients were lower than those elicited in neuromyelitis optica spectrum disorders (NMOSDs) and in healthy subjects (Cossu et al., 2014, 2017b). Intracerebral infections with BCG were capable of suppressing immune responses and reducing the severity of EAE in mice (Lee et al., 2008). Additionally, active infection by intraperitoneal injections with live BCG in EAE-induced mice reduced the MOG_35-55_-specific cells numbers in the CNS through diverting the traffic of autoreactive T cells to local BCG inflammatory sites (Sewell et al., 2003). Clinical trials showed a beneficial effect of BCG vaccination in patients with relapsing-remitting MS, with reduced magnetic resonance imaging (MRI) activity (Ristori et al., 2014). These observations imply a protective role of the BCG vaccine against MS progression, but its precise mechanisms remain unclear.

The innate immune response to the BCG vaccine initiates at the site of inoculation, where APCs of the skin phagocytose and degrade the bacillus by intracellular killing mechanisms. Peptides are presented to cells of the adaptive immune system by MHC class I and class II proteins.

The BCG vaccine strongly induces Th1 and Th17 effector cells, and regulatory T cells following vaccination (Moliva et al., 2017). Further, the vaccine induces a persistent immunological memory for mycobacterial antigens for several years (Ben-Smith et al., 2008). Therefore, it is possible that BCG vaccination modulates the initiation and/or progression of MS, essentially a CD4+ T cell-mediated disease with Th1, Th17, and Th9 phenotypes. Concerning the Th9 subset, although the pathogenic or non-pathogenic function of IL-9 in CNS inflammation remains to be clarified, evidence suggests that Th9 cells play an important role in MS (Elyaman and Khoury, 2017). Findings from a recent study revealed that TLR2 co-stimulation with the *Mycobacterium tuberculosis* Ag85B antigen significantly increased IL-9 secretion from both mouse and human CD4+ T cells (Karim et al., 2017).

The contrasting roles of BCG and MAP in the etiopathogenesis of MS may be due to the different routes of antigen transmission/administration (intradermal for BCG and oral for MAP) and the antigen-specific immune responses to mycobacterial components. For instance, the *Mycobacterium tuberculosis* Ag85B peptide shows 100% identity to BCG, but does not share significant sequence similarity with MAP. The MAP lipopentapeptide, a cell wall component capable of inducing specific humoral and cell-mediated immune responses in cattle, is unique to MAP and is absent from the cell wall of other mycobacterial species (Cossu et al., 2017b).

Human and animal research have demonstrated that γδ T cells play an important role in anti-mycobacterial immunity, including in the mechanisms of BCG immunization or in MAP-infected calves (Chen, 2005). Like iNKT cells, γδ T cells did not require MHC recognition and they recognized mycobacterial lipid antigens presented by CD1 molecules (Chen, 2005). These cells are the major source of IL9 in humans (Peters et al., 2016). Although the relationship between Th9 cells and mycobacteria remains unknown, γδ T cells may have a distinct role in generating a primary immune response to certain pathogens.

## Chlamydia

*Chlamydia pneumoniae*, a common cause of human respiratory diseases, is an obligate intracellular microorganism that inhibits host cell apoptosis to escape from CD4+ and CD8+ immune recognition (Kun et al., 2013). During infection, TLR2 and TLR4 signaling are important for the initial host defense against bacterial lipopolysaccharide (LPS) and heat shock proteins, whereas TLR3 and TLR9 are mainly responsible for recognizing microbial nucleic acids in mice (Shimada et al., 2012). Nod1 and Nod2 respond to intracellular fragments of bacterial peptidoglycans and play a role in sequential intracellular events by prolonging the activation of target cells by intracellular bacteria in humans (Halme et al., 2000).

*Chlamydia pneumoniae* is capable of infecting neuronal glia and neuronal ependymal cells within the CNS in mice (Du et al., 2002) and has been reported to cross the BBB, altering its permeability (MacIntyre et al., 2002). The bacterium can induce a persistent infection in the brain and consequently has been related to several chronic inflammatory diseases, such as MS and Alzheimer’s disease (Kern et al., 2009). The association with *Chlamydia pneumoniae* and MS was intensively investigated with controversial results and remains a matter of debate.

Molecular and seroprevalence studies revealed a higher presence of intrathecal antibodies of *Chlamydia pneumoniae* in the CSF of subgroups of MS patients compared to healthy controls (Fainardi et al., 2008; Ivanova et al., 2015); however, these antibodies were also found in other inflammatory neurological conditions such as NMOSDs (Yoshimura et al., 2013), another type of inflammatory disease of the CNS that shares features with MS.

Culturing these neurotropic bacteria from CSF and MS brain lesions, as well as molecular detection by PCR are difficult due to the absence of a standardized detection protocol. A study showed that qualitative colorimetric microtiter plate-based PCR–enzyme immunoassay (PCR–EIA) and nested PCR are the most sensitive detection methods for bacterial DNA in the CSF of MS patients, although no evidence of recent *Chlamydia pneumoniae* infection in the brain was found (Tang et al., 2009). However, infection by the live bacterium can worsen EAE in mice (Du et al., 2002).

In multiple clinical trials conducted to assess the effect of treatment with rifampin and azithromycin against *Chlamydia*, MS patients did not show any beneficial outcome in gadolinium-enhancing lesions, relapse rates, and disease severity (Sriram et al., 2005). However, compared with patients treated with an antibiotic, placebo controls showed a significant decrease in brain parenchymal volume, a marker of global brain atrophy (Sriram et al., 2005). These data suggest that *Chlamydia pneumoniae* or Chlamydia-like organisms might not be causative agents of MS (Contini et al., 2008), but could act as a co-factor in the development of the disease.

## Helicobacter

*Helicobacter pylori* colonizes the surface of gastric epithelial cells and is generally considered a non-invasive bacterium, but *in vitro* observations have demonstrated that it can enter host epithelial and immune cells (Weir et al., 2010). The bacterium infects over 50% of the population worldwide and a relationship with MS has been reported (Jaruvongvanich et al., 2016); however, its potential role in the disease is unclear and controversial.

In agreement with the hygiene hypothesis, which assumes that infections during childhood are essential for preventing autoimmune conditions later in life, immunity to *Helicobacter pylori* seems to protect against the development of MS. Indeed, meta-analyses have shown that the bacterial presence and MS are negatively correlated in western countries (Jaruvongvanich et al., 2016; Yao et al., 2016). Concerning Asian countries, antibodies against *Helicobacter pylori* antigens were more prevalent in aquaporin 4 antibody-positive NMOSD patients, but negative in MS patients (Yoshimura et al., 2013). Furthermore, *Helicobacter pylori* infection seems to exert some protective role against EAE, inhibiting both Th1 and Th17 responses and reducing the severity of the disease (Cook et al., 2015).

*Helicobacter pylori* can evade pathogen recognition by the innate immune system by manipulating PRRs (such as TLR4, which recognizes LPS), thereby using the immune system to induce an anti-inflammatory response (Efthymiou et al., 2017). In addition, *Helicobacter* antigens can also inhibit activation of the adaptive immune system by suppressing Th1/Th17 cell responses by FoxP3(+) regulatory T cells (Salama et al., 2013). These findings support a protective role of this gram-positive bacterium in autoimmune diseases such as MS.

While these data do not suggest any link between the bacteria and MS susceptibility, a previous report showed a high frequency of acute *Helicobacter pylori* infection in 44 relapsing–remitting MS patients in stable phase (Gavalas et al., 2015). Instead, we showed a lack of humoral responses against the *Helicobacter pylori* HP986 protein, previously associated with peptic ulcer and gastric carcinoma, in a cohort of 119 MS patients from Sardinia (80.5% relapsing–remitting in acute phase) (Cossu et al., 2012).

In a recent seroprevalence study performed in Greece, antibodies against to the vacuolating cytotoxin A antigen of *Helicobacter pylori*, a virulence factor involved in gastric injury, were detected more frequently in secondary progressive MS patients compare to healthy controls, suggesting that antigen recognition by serum antibodies differed not only between patients and controls, but also amongst patients with relapsing–remitting and secondary progressive MS (Efthymiou et al., 2017).

Persistent bacterial infection may cause a loss of self-tolerance due to the constant release of bacterial antigens able to stimulate the release of pro-inflammatory cytokines from immune cells. *Helicobacter pylori* can exert these effects not only locally, but also directly via the CNS with modulation of the brain–gut axis (Kountouras et al., 2015).

## Mycoplasma

Another possible co-factor in MS can be *Mycoplasma pneumoniae*, a small prokaryotic organism that adheres to host cells by specialized attachments and adhesin proteins, but which also possesses cellular invasive capacity (Waites and Talkington, 2004).

The mycoplasma lipoproteins play a key role in infection and modulate immunity via TLR1 and TLR2 (Waites and Talkington, 2004). Co-expression of TLR2 and TLR6 mediates cellular response to lipopeptides from *Mycoplasma pneumoniae*, it has been identified in cerebral endothelial cells and microglia of MS patients by RT-PCR (Bsibsi et al., 2002). A TLR6 polymorphism was also associated with the development of interferon-beta specific neutralizing antibodies in male MS patients (Enevold et al., 2010).

In a study conducted on 130 relapsing–remitting MS Iranian patients, mycoplasma IgG and IgM antibodies were more prevalent in woman patients than in control subjects, but seropositivity was not significantly different during the various phases of disease activity compared to controls (Bahar et al., 2012). MS is more prevalent in women, suggesting differences in the immune system between women and men, which might be caused by different factors like sex hormones or by a different genetic predisposition to infection (Harbo et al., 2013). For this reason, gender differences might influence the susceptibility to chronic infections caused by *Mycoplasma pneumoniae*, which might play a role in the development of MS, at least among women.

Tetracycline-resistant mutated *mycoplasma* was detected by nested-PCR in four urine samples collected from patients with MS (Naghib et al., 2017). Conversely, two different studies failed to find evidence for the presence of bacteria in the CSF and serum from patients diagnosed with MS by targeting the *Mycoplasma* 16S rDNA gene (Casserly et al., 2007; Lindsey and Patel, 2008). A case report showed that the bacterium is capable of invading the CNS (Abramovitz et al., 1987); therefore, we cannot exclude the possibility that mycoplasma infection can contribute to disease-inducing mechanisms, resulting in CNS autoimmunity. Despite these conflicting findings, many important issues remain unanswered regarding the influence of *Mycoplasma pneumonia* on MS.

## Clostridia

*Clostridium perfringens* is an anaerobic gut bacterium that causes necrotic enteritis in poultry and occasionally can cause foodborne illness outbreaks in humans (Lu et al., 2009). TLR2 is involved in the host response to *Clostridium perfringens* infection in chickens (Lu et al., 2009). *Clostridium perfringens* is classified into five strain types based on the toxins produced, and type B releases epsilon toxin, which causes adverse effects in the CNS (Rumah et al., 2015).

Epsilon toxin is absorbed in the intestines in mice and can permeabilize the BBB (Rumah et al., 2015). Animal studies revealed the toxin can bind to retinal vessels that form the blood–retinal barrier, and it also has acute effects on neuronal conductivity in mouse optic nerves (Cases et al., 2017). Visual impairment is one of the most common initial clinical manifestations of MS (Sakai et al., 2011). Epsilon toxin can specifically bind to CNS endothelial cells, myelinated fibers, and oligodendrocytes in mammals (Dorca-Arévalo et al., 2008).

*Clostridium perfringens* type B was isolated for the first time from a fecal sample of a patient with initial clinical symptoms of MS and with active enhancing lesions found on a brain MRI (Rumah et al., 2013). In addition, increased immunoreactivity to epsilon toxin in sera and CSF from patients with MS was observed (compared to healthy controls), suggesting a prior exposure to the toxin in the MS population (Rumah et al., 2013). Taken together, these data support the hypothetical contribution of the *Clostridium perfringens* type B epsilon toxin in triggering newly formed MS lesions.

Interestingly, in the same work, the presence of *Clostridium perfringens* type A, a pathogenic bacterium that causes food poisoning and gas gangrene, was lower in the gut of MS patients compared to healthy controls, but in overabundance in NMOSD (Rumah et al., 2013). This commensal bacterium could also contribute to NMOSD pathogenesis. Indeed, T cells recognizing the immunodominant T cell epitope of aquaporin-4, the autoantigen associated with NMOSD, exhibited cross-reactivity to a homologous peptide belonging to a *Clostridium perfringens* protein (Zamvil et al., 2018). This finding does not necessarily imply a protective role of *Clostridia* in MS; however, pathogenic bacteria could also be beneficial under certain conditions resulting from a complex interplay between gut microbiota, host genetics, and diet.

## Borrelia

*Borrelia burgdorferi* is the etiological agent of Lyme disease, a syndrome resembling MS. The bacterium can induce TLR2-dependent macrophage activation and can drive Th1-type T-cell immunity (Whiteside et al., 2018). During an infection, *Borrelia burgdorferi* employs different mechanisms to manipulate the innate and adaptive immune systems, in order to survive inside mammalian host cells (Tracy and Baumgarth, 2017). A study conducted in Poland on 769 neurological patients showed that 38.5% of MS subjects were positive for *Borrelia burgdorferi* antibodies, indicating that MS may often be associated with *Borrelia* infection (Chmielewska-Badora et al., 2000). In contrast, data from a study showed the presence of antibodies in 14.2% of 106 MS patients versus 25.3% of 103 healthy controls (Schmutzhard et al., 1988), while in another study performed in an endemic area for Lyme disease, only 1 out of 89 MS patients was antibody-positive (Coyle, 1989). In conclusion, because Lyme disease mimics several neurologic symptoms of MS, it is an important disease to test for during differential diagnosis of MS. Moreover, the presence of antibodies against *Borrelia burgdorferi* in MS patients does not enable distinction between past/current infections, or prove that the bacterium is the cause of the disease.

## Gut Microbiota

The major role of the human gut microbiota is to regulate innate and adaptive immune homeostasis, and the dynamic crosstalk between the host and its microbiota is mediated by the recognition of conserved microbe-associated molecular patterns (Rakoff-Nahoum et al., 2004).

The microbiome interacts with the CNS though bidirectional signaling from the gut microbiota to the brain, and vice versa (Carabotti et al., 2015). Microbiota can interact with the gut–brain axis though different mechanisms, including modulation of the intestinal barrier, or at level of the BBB by translocating or releasing factors into the bloodstream (Logsdon et al., 2018).

Gut microbial dysbiosis has been associated with MS; however, exactly how the host immune system is influenced by alteration of the microbiota is not clear. The mesenteric lymphatic vessels play a role in immune cells and metabolism trafficking, such as the induction of regulatory cells, which are directly responsible for suppressing autoreactive T cells that infiltrate into the CNS (Colpitts and Kasper, 2017). Abnormalities in the microbiome may influence the balance between cells driving disease such as Th1/Th17, Th2, Th9, and regulatory cells, and can be critical in the development of inflammatory and immune processes.

According to most microbiome studies in patients with MS, there is a lower abundance or depletion of bacteria capable of inducing immuno-regulatory cells to limit inflammation, such as *Clostridia*, *Bacteroides*, or *Actinobacteria* (Tremlett and Waubant, 2017). Conversely, compared to healthy subjects, patients with MS seemed to have a higher abundance of bacteria with the ability to trigger pro-inflammatory host responses, such as *Firmicutes*, *Euryarcheaeota* (Shahi et al., 2017) and *Proteobacteria* (Shahi et al., 2017). In a pilot study conducted on 17 patients with relapsing–remitting pediatric MS, a shorter time to relapse was associated with a depletion of *Fusobacteria* and an expansion of *Firmicutes* (Tremlett et al., 2016).

A recent study of identical twins discordant for MS showed that transfer of the gut microbiota from an MS-affected twin into transgenic mice expressing a myelin autoantigen-specific TCR induced a higher incidence of autoimmunity, compared to the transfer of healthy twin microbiota (Berer et al., 2017). The most significant difference was a relative abundance of the *Sutterella* genus in fecal samples of mice that received the microbiota from healthy donor, compared to mice colonized by MS twins.

The microbiome of patients with MS differs from that of healthy subjects, but it remains unknown whether the altered microbiota is a cause or consequence of the development of MS. Therefore, prospective cohort studies on the effects of host microbiota on MS patients are needed.

## Conclusion

In 1890, Robert Koch formulated postulates for determining that a particular bacterium is the cause of a specific disease. According to these criteria, the causative organism must be found and isolated in every case of the disease and absent in the healthy subjects. Despite the importance of these postulates in the development of microbiology, there are many limitations associated with them, and with the advent of new molecular and genetic techniques in the fields of microbiology and medicine, these criteria of causation have been revised several times. Nevertheless, these criteria for infectious disease causality are still considered of contemporary relevance and might still have some use.

To date, none of the bacteria related to MS have fulfilled Koch’s postulates, and a causative relationship between a specific bacterium or vaccination with a live attenuated organism and MS has yet to be established. Current data suggest that multiple infections along with non-infectious environmental factors might trigger the development of MS in a certain genetic background. It is possible that a single bacterium need not be responsible for MS, but different pathogens may initiate events that trigger a common immune-pathologic pathway. In addition, an infectious pathogen may not be causative, but it may still influence the development and progression of MS, having a protective role or exacerbating disease manifestation during immunological maturation.

Interestingly, the existence of an abnormal immunological mechanism has been shown to operate in the pre-disease stage of MS, which in turn favors the phenomenon of epitope spreading (Achiron et al., 2010). Considering that the time interval between a bacterial infection and MS initiation may not be immediate and that a long period between the two events may be needed, bacteria could be one of several environmental factors responsible for the activation of this apoptotic process in a time-dependent mechanism.

Another issue is that most pathogens related to MS, with few exceptions, seem to be highly prevalent in the general population; however, the increase of MS incidence is not ubiquitous and depends on various environmental and/or genetic factors such as latitude, ethnicity, and development of the country.

Future research should concentrate on combining data obtained from animal models and epidemiological studies, in order to better explain specific aspects of host–pathogen interactions and, consequently, define the role of bacteria in the etiology and pathogenesis of MS.

## Author Contributions

DC performed the experiments related to several articles included in this review and wrote the manuscript. KY and NH wrote the manuscript and supported in the critical reading.

## Conflict of Interest Statement

KY and NH research studies are financially supported by Ohara Pharmaceutical, AbbVie, Ono Pharmaceutical, Mitsubishi Tanabe Pharma, MiZ, Asahi Kasei Medical, Nihon Pharmaceutical. The remaining author declares that the research was conducted in the absence of any commercial or financial relationships that could be construed as a potential conflict of interest.

## References

[B1] Abramovitz P. Schvartzman P. Harel D. Lis I. Naot Y. (1987). Direct invasion of the central nervous system by *Mycoplasma pneumoniae*: a report of two cases. *J. Infect. Dis.* 155 482–487. 10.1093/infdis/155.3.482 3100660

[B2] Achiron A. Grotto I. Balicer R. Magalashvili D. Feldman A. Gurevich M. (2010). Microarray analysis identifies altered regulation of nuclear receptor family members in the pre-disease state of multiple sclerosis. *Neurobiol. Dis.* 38 201–209. 10.1016/j.nbd.2009.12.029 20079437

[B3] Akira S. Uematsu S. Takeuchi O. (2006). Pathogen recognition and innate immunity. *Cell* 124 783–801. 10.1016/j.cell.2006.02.015 16497588

[B4] Almand E. A. Moore M. D. Jaykus L. A. (2017). Virus-bacteria interactions: an emerging topic in human infection. *Viruses* 9:E58. 10.3390/v9030058 28335562 PMC5371813

[B5] Arsenault R. J. Li Y. Maattanen P. Scruten E. Doig K. Potter A. (2013). Altered Toll-like receptor 9 signaling in *Mycobacterium avium* subsp. *paratuberculosis*-infected bovine monocytes reveals potential therapeutic targets. *Infect. Immun.* 81 226–237. 10.1128/IAI.00785-12 23115040 PMC3536146

[B6] Arsenault R. J. Maattanen P. Daigle J. Potter A. Griebel P. Napper S. (2014). From mouth to macrophage: mechanisms of innate immune subversion by *Mycobacterium avium* subsp. *paratuberculosis*. *Vet. Res.* 45:54. 10.1186/1297-9716-45-54 24885748 PMC4046017

[B7] Ascherio A. Munger K. L. (2010). 99th Dahlem conference on infection, inflammation and chronic inflammatory disorders: Epstein-Barr virus and multiple sclerosis: epidemiological evidence. *Clin. Exp. Immunol.* 160 120–124. 10.1111/j.1365-2249.2010.04121.x 20415861 PMC2841845

[B8] Bahar M. Ashtari F. Aghaei M. Akbari M. Salari M. Ghalamkari S. (2012). *Mycoplasma pneumonia* seroposivity in Iranian patients with relapsing-remitting multiple sclerosis: a randomized case-control study. *J. Pak. Med. Assoc.* 62 S6–S8. 22768448

[B9] Ben-Smith A. Fine P. E. Dockrell H. M. (2008). Persistence of the immune response induced by BCG vaccination. *BMC Infect. Dis.* 8:9. 10.1186/1471-2334-8-9 18221509 PMC2263052

[B10] Berer K. Gerdes L. A. Cekanaviciute E. Jia X. Xiao L. Xia Z. (2017). Gut microbiota from multiple sclerosis patients enables spontaneous autoimmune encephalomyelitis in mice. *Proc. Natl. Acad. Sci. U.S.A.* 114 10719–10724. 10.1073/pnas.1711233114 28893994 PMC5635914

[B11] Bettelli E. Pagany M. Weiner H. L. Linington C. Sobel R. A. Kuchroo V. K. (2003). Myelin oligodendrocyte glycoprotein-specific T cell receptor transgenic mice develop spontaneous autoimmune optic neuritis. *J. Exp. Med.* 197 1073–1081. 10.1084/jem.20021603 12732654 PMC2193967

[B12] Billiau A. Matthys P. (2001). Modes of action of Freund’s adjuvants in experimental models of autoimmune diseases. *J. Leukoc. Biol.* 70 849–860. 11739546

[B13] Brocke S. Gaur A. Piercy C. Gautam A. Gijbels K. Fathman C. G. (1993). Induction of relapsing paralysis in experimental autoimmune encephalomyelitis by bacterial superantigen. *Nature* 365 642–644. 10.1038/365642a0 7692305

[B14] Bsibsi M. Ravid R. Gveric D. van Noort J. M. (2002). Broad expression of Toll-like receptors in the human central nervous system. *J. Neuropathol. Exp. Neurol.* 61 1013–1021. 10.1093/jnen/61.11.1013 12430718

[B15] Carabotti M. Scirocco A. Maselli M. A. Severi C. (2015). The gut-brain axis: interactions between enteric microbiota, central and enteric nervous systems. *Ann. Gastroenterol.* 28 203–209. 25830558 PMC4367209

[B16] Cases M. Llobet A. Terni B. Gómez de Aranda I. Blanch M. Doohan B. (2017). Acute effect of pore-forming *Clostridium perfringens* ε-toxin on compound action potentials of optic nerve of mouse. *eNeuro* 4:ENEURO.0051-17.2017. 10.1523/ENEURO.0051-17.2017 28798954 PMC5550839

[B17] Casserly G. Barry T. Tourtellotte W. W. Hogan E. L. (2007). Absence of Mycoplasma-specific DNA sequence in brain, blood and CSF of patients with multiple sclerosis (MS): a study by PCR and real-time PCR. *J. Neurol. Sci.* 253 48–52. 10.1016/j.jns.2006.11.017 17234214

[B18] Chen Z. W. (2005). Immune regulation of gammadelta T cell responses in mycobacterial infections. *Clin. Immunol.* 116 202–207. 10.1016/j.clim.2005.04.005 16087145 PMC2869281

[B19] Chmielewska-Badora J. Cisak E. Dutkiewicz J. (2000). Lyme borreliosis and multiple sclerosis: any connection? A seroepidemic study. *Ann. Agric. Environ. Med.* 7 141–143. 11153045

[B20] Colpitts S. L. Kasper L. H. (2017). Influence of the gut microbiome on autoimmunity in the central nervous system. *J. Immunol.* 198 596–604. 10.4049/jimmunol.1601438 28069755

[B21] Contini C. Seraceni S. Cultrera R. Castellazzi M. Granieri E. Fainardi E. (2008). Molecular detection of Parachlamydia-like organisms in cerebrospinal fluid of patients with multiple sclerosis. *Mult. Scler.* 14 564–566. 10.1177/1352458507085796 18562511

[B22] Cook K. W. Crooks J. Hussain K. O’Brien K. Braitch M. Kareem H. (2015). *Helicobacter pylori* infection reduces disease severity in an experimental model of multiple sclerosis. *Front. Microbiol.* 6:52. 10.3389/fmicb.2015.00052 25762984 PMC4327743

[B23] Correale J. Farez M. F. (2012). Does helminth activation of toll-like receptors modulate immune response in multiple sclerosis patients? *Front. Cell. Infect. Microbiol.* 2:112. 10.3389/fcimb.2012.00112 22937527 PMC3426839

[B24] Cosorich I. Dalla-Costa G. Sorini C. Ferrarese R. Messina M. J. Dolpady J. (2017). High frequency of intestinal TH17 cells correlates with microbiota alterations and disease activity in multiple sclerosis. *Sci. Adv.* 3:e1700492. 10.1126/sciadv.1700492 28706993 PMC5507635

[B25] Cossu D. Cocco E. Paccagnini D. Masala S. Ahmed N. Frau J. (2011). Association of *Mycobacterium avium* subsp. *paratuberculosis* with multiple sclerosis in Sardinian patients. *PLoS One* 6:e18482. 10.1371/journal.pone.0018482 21533236 PMC3076380

[B26] Cossu D. Mameli G. Galleri G. Cocco E. Masala S. Frau J. (2015). Human interferon regulatory factor 5 homologous epitopes of Epstein-Barr virus and *Mycobacterium avium* subsp. *paratuberculosis* induce a specific humoral and cellular immune response in multiple sclerosis patients. *Mult. Scler.* 21 984–995. 10.1177/1352458514557304 25392335

[B27] Cossu D. Mameli G. Masala S. Cocco E. Frau J. Marrosu M. G. (2014). Evaluation of the humoral response against mycobacterial peptides, homologous to MOG35^−^55, in multiple sclerosis patients. *J. Neurol. Sci.* 347 78–81. 10.1016/j.jns.2014.09.023 25271190

[B28] Cossu D. Masala S. Cocco E. Paccagnini D. Frau J. Marrosu M. G. (2012). Are *Mycobacterium avium* subsp. paratuberculosis and Epstein-Barr virus triggers of multiple sclerosis in Sardinia? *Mult. Scler.* 18 1181–1184. 10.1177/1352458511433430 22261119

[B29] Cossu D. Masala S. Sechi L. A. (2013). A Sardinian map for multiple sclerosis. *Future Microbiol.* 8 223–232. 10.2217/fmb.12.135 23374127

[B30] Cossu D. Yokoyama K. Hattori N. (2017a). Conflicting role of *Mycobacterium* species in multiple sclerosis. *Front. Neurol.* 8:216. 10.3389/fneur.2017.00216 28579973 PMC5437105

[B31] Cossu D. Yokoyama K. Tomizawa Y. Momotani E. Hattori N. (2017b). Altered humoral immunity to mycobacterial antigens in Japanese patients affected by inflammatory demyelinating diseases of the central nervous system. *Sci. Rep.* 7:3179. 10.1038/s41598-017-03370-z 28600575 PMC5466620

[B32] Coyle P. K. (1989). *Borrelia burgdorferi* antibodies in multiple sclerosis patients. *Neurology* 39 760–761. 10.1212/WNL.39.6.760 2725867

[B33] Dando S. J. Mackay-Sim A. Norton R. Currie B. J. St John J. A. Ekberg J. A. (2014). Pathogens penetrating the central nervous system: infection pathways and the cellular and molecular mechanisms of invasion. *Clin. Microbiol. Rev.* 27 691–726. 10.1128/CMR.00118-13 25278572 PMC4187632

[B34] Doran K. S. Banerjee A. Disson O. Lecuit M. (2013). Concepts and mechanisms: crossing host barriers. *Cold Spring Harb. Perspect. Med.* 3:a010090. 10.1101/cshperspect.a010090 23818514 PMC3685877

[B35] Dorca-Arévalo J. Soler-Jover A. Gibert M. Popoff M. R. Martín-Satué M. Blasi J. (2008). Binding of epsilon-toxin from *Clostridium perfringens* in the nervous system. *Vet. Microbiol.* 131 14–25. 10.1016/j.vetmic.2008.02.015 18406080

[B36] Du C. Yao S. Y. Ljunggren-Rose A. Sriram S. (2002). *Chlamydia pneumoniae* infection of the central nervous system worsens experimental allergic encephalitis. *J. Exp. Med.* 196 1639–1644. 10.1084/jem.20020393 12486106 PMC2196067

[B37] Efthymiou G. Dardiotis E. Liaskos C. Marou E. Tsimourtou V. Rigopoulou E. I. (2017). Immune responses against *Helicobacter pylori*-specific antigens differentiate relapsing remitting from secondary progressive multiple sclerosis. *Sci. Rep.* 7:7929. 10.1038/s41598-017-07801-9 28801580 PMC5554191

[B38] Elyaman W. Khoury S. J. (2017). Th9 cells in the pathogenesis of EAE and multiple sclerosis. *Semin. Immunopathol.* 39 79–87. 10.1007/s00281-016-0604-y 27844107

[B39] Enevold C. Oturai A. B. Sorensen P. S. Ryder L. P. Koch-Henriksen N. Bendtzen K. (2010). Polymorphisms of innate pattern recognition receptors, response to interferon-beta and development of neutralizing antibodies in multiple sclerosis patients. *Mult. Scler.* 16 942–949. 10.1177/1352458510373264 20595247

[B40] Fainardi E. Castellazzi M. Seraceni S. Granieri E. Contini C. (2008). Under the microscope: focus on *Chlamydia pneumoniae* infection and multiple sclerosis. *Curr. Neurovasc. Res.* 5 60–70. 10.2174/156720208783565609 18289023

[B41] Fleming J. Fabry Z. (2007). The hygiene hypothesis and multiple sclerosis. *Ann. Neurol.* 61 85–89. 10.1002/ana.21092 17315205

[B42] Floreani A. Leung P. S. Gershwin M. E. (2016). Environmental basis of autoimmunity. *Clin. Rev. Allergy Immunol.* 50 287–300. 10.1007/s12016-015-8493-8 25998909

[B43] Ganong W. F. (2000). *Circumventricular organs*: definition and role in the regulation of endocrine and autonomic function. *Clin. Exp. Pharmacol. Physiol.* 27 422–427. 10.1046/j.1440-1681.2000.03259.x 10831247

[B44] Gao Z. Lv J. Wang M. (2017). Epstein-Barr virus is associated with periodontal diseases: a meta-analysis based on 21 case-control studies. *Medicine* 96:e5980. 10.1097/MD.0000000000005980 28178139 PMC5312996

[B45] Gavalas E. Kountouras J. Boziki M. Zavos C. Polyzos S. A. Vlachaki E. (2015). Relationship between *Helicobacter pylori* infection and multiple sclerosis. *Ann. Gastroenterol.* 28 353–356. 26126617 PMC4480172

[B46] Gharagozloo M. Gris K. V. Mahvelati T. Amrani A. Lukens J. R. Gris D. (2018). NLR-dependent regulation of inflammation in multiple sclerosis. *Front. Immunol.* 8:2012. 10.3389/fimmu.2017.02012 29403486 PMC5778124

[B47] Halme S. Latvala J. Karttunen R. Palatsi I. Saikku P. Surcel H. M. (2000). Cell-mediated immune response during primary *Chlamydia pneumoniae* infection. *Infect. Immun.* 68 7156–7158. 10.1128/IAI.68.12.7156-7158.2000 11083846 PMC97831

[B48] Harbo H. F. Gold R. Tintoré M. (2013). Sex and gender issues in multiple sclerosis. *Ther. Adv. Neurol. Disord.* 6 237–248. 10.1177/1756285613488434 23858327 PMC3707353

[B49] Hofstetter H. H. Shive C. L. Forsthuber T. G. (2002). Pertussis toxin modulates the immune response to neuroantigens injected in incomplete Freund’s adjuvant: induction of Th1 cells and experimental autoimmune encephalomyelitis in the presence of high frequencies of Th2 cells. *J. Immunol.* 169 117–125. 10.4049/jimmunol.169.1.117 12077236

[B50] Ivanova M. V. Kolkova N. I. Morgunova E. Y. Pashko Y. P. Zigangirova N. A. Zakharova M. N. (2015). Role of Chlamydia in multiple sclerosis. *Bull. Exp. Biol. Med.* 159 646–648. 10.1007/s10517-015-3037-z 26468024

[B51] Jaruvongvanich V. Sanguankeo A. Jaruvongvanich S. Upala S. (2016). Association between *Helicobacter pylori* infection and multiple sclerosis: a systematic review and meta-analysis. *Mult. Scler. Relat. Disord.* 7 92–97. 10.1016/j.msard.2016.03.013 27237767

[B52] Karim A. F. Reba S. M. Li Q. Boom W. H. Rojas R. E. (2017). Toll like Receptor 2 engagement on CD4^+^ T cells promotes TH9 differentiation and function. *Eur. J. Immunol.* 47 1513–1524. 10.1002/eji.201646846 28665005 PMC5606324

[B53] Kern J. M. Maass V. Maass M. (2009). Molecular pathogenesis of chronic *Chlamydia pneumoniae* infection: a brief overview. *Clin. Microbiol. Infect.* 15 36–41. 10.1111/j.1469-0691.2008.02631.x 19220338

[B54] Kountouras J. Zavos C. Polyzos S. A. Deretzi G. (2015). The gut-brain axis: interactions between *Helicobacter pylori* and enteric and central nervous systems. *Ann. Gastroenterol.* 28:506. 26423130 PMC4585404

[B55] Kreiss M. Asmuss A. Krejci K. Lindemann D. Miyoshi-Akiyama T. Uchiyama T. (2004). Contrasting contributions of complementarity-determining region 2 and hypervariable region 4 of rat BV8S2+ (Vbeta8.2) TCR to the recognition of myelin basic protein and different types of bacterial superantigens. *Int. Immunol.* 16 655–663. 10.1093/intimm/dxh068 15096488

[B56] Kun D. Xiang-Lin C. Ming Z. Qi L. (2013). Chlamydia inhibit host cell apoptosis by inducing Bag-1 via the MAPK/ERK survival pathway. *Apoptosis* 18 1083–1092. 10.1007/s10495-013-0865-z 23708800

[B57] Kurtzke J. F. Heltberg A. (2001). Multiple sclerosis in the Faroe Islands: an epitome. *J. Clin. Epidemiol.* 54 1–22. 10.1016/S0895-4356(00)00268-7 11165464

[B58] Lee J. Reinke E. K. Zozulya A. L. Sandor M. Fabry Z. (2008). *Mycobacterium bovis* bacille Calmette-Guérin infection in the CNS suppresses experimental autoimmune encephalomyelitis and Th17 responses in an IFN-gamma-independent manner. *J. Immunol.* 181 6201–6212. 10.4049/jimmunol.181.9.6201 18941210 PMC2735452

[B59] Li Y. Huang Y. Lue J. Quandt J. A. Martin R. Mariuzza R. A. (2005). Structure of a human autoimmune TCR bound to a myelin basic protein self-peptide and a multiple sclerosis-associated MHC class II molecule. *EMBO J.* 24 2968–2979. 10.1038/sj.emboj.7600771 16079912 PMC1201352

[B60] Lindsey J. Patel S. (2008). PCR for bacterial 16S ribosomal DNA in multiple sclerosis cerebrospinal fluid. *Mult. Scler.* 14 147–152. 10.1177/1352458507082149 17986505

[B61] Logsdon A. F. Erickson M. A. Rhea E. M. Salameh T. S. Banks W. A. (2018). Gut reactions: how the blood-brain barrier connects the microbiome and the brain. *Exp. Biol. Med.* 243 159–165. 10.1177/1535370217743766 29169241 PMC5788145

[B62] Louveau A. Harris T. H. Kipnis J. (2015). Revisiting the mechanisms of CNS immune privilege. *Trends Immunol.* 36 569–577. 10.1016/j.it.2015.08.006 26431936 PMC4593064

[B63] Lu Y. Sarson A. J. Gong J. Zhou H. Zhu W. Kang Z. (2009). Expression profiles of genes in Toll-like receptor-mediated signaling of broilers infected with *Clostridium perfringens*. *Clin. Vaccine Immunol.* 16 1639–1647. 10.1128/CVI.00254-09 19776194 PMC2772368

[B64] MacIntyre A. Hammond C. J. Little C. S. Appelt D. M. Balin B. J. (2002). *Chlamydia pneumoniae* infection alters the junctional complex proteins of human brain microvascular endothelial cells. *FEMS Microbiol. Lett.* 217 167–172. 10.1111/j.1574-6968.2002.tb11470.x 12480099

[B65] Mameli G. Cocco E. Frau J. Marrosu M. G. Sechi L. A. (2016). Epstein Barr Virus and *Mycobacterium avium* subsp. *paratuberculosis* peptides are recognized in sera and cerebrospinal fluid of MS patients. *Sci. Rep.* 6:22401. 10.1038/srep22401 26956729 PMC4783662

[B66] Mameli G. Cossu D. Cocco E. Masala S. Frau J. Marrosu M. G. (2014). Epstein-Barr virus and *Mycobacterium avium* subsp. *paratuberculosis* peptides are cross recognized by anti-myelin basic protein antibodies in multiple sclerosis patients. *J. Neuroimmunol.* 270 51–55. 10.1016/j.jneuroim.2014.02.013 24642384

[B67] Maver A. Lavtar P. Ristiæ S. Stopinšek S. Simèiè S. Hoèevar K. (2017). Identification of rare genetic variation of NLRP1 gene in familial multiple sclerosis. *Sci. Rep.* 7:3715. 10.1038/s41598-017-03536-9 28623311 PMC5473861

[B68] Miranda-Hernandez S. Baxter A. G. (2013). Role of toll-like receptors in multiple sclerosis. *Am. J. Clin. Exp. Immunol.* 2 75–93. 23885326 PMC3714200

[B69] Moliva J. I. Turner J. Torrelles J. B. (2017). Immune responses to Bacillus Calmette-Guérin vaccination: why do they fail to protect against *Mycobacterium tuberculosis*? *Front. Immunol.* 8:407. 10.3389/fimmu.2017.00407 28424703 PMC5380737

[B70] Naghib M. Kheirkhah B. Mohebbi R. Sadeg L. (2017). Molecular identification of drug resistant mutations to tetracycline in *Mycoplasma* spp. isolated from patients with multiple sclerosis. *Cell. Mol. Biol.* 63 112–115. 10.14715/cmb/2017.63.7.19 28838350

[B71] Oldstone M. B. (2014). Molecular mimicry: its evolution from concept to mechanism as a cause of autoimmune diseases. *Monoclon. Antib. Immunodiagn. Immunother.* 33 158–165. 10.1089/mab.2013.0090 24694269 PMC4063373

[B72] Olsson T. Barcellos L. F. Alfredsson L. (2017). Interactions between genetic, lifestyle and environmental risk factors for multiple sclerosis. *Nat. Rev. Neurol.* 13 25–36. 10.1038/nrneurol.2016.187 27934854

[B73] Pawlowski A. Jansson M. Sköld M. Rottenberg M. E. Källenius G. (2012). Tuberculosis and HIV co-infection. *PLoS Pathog.* 8:e1002464. 10.1371/journal.ppat.1002464 22363214 PMC3280977

[B74] Peters C. Häsler R. Wesch D. Kabelitz D. (2016). Human Vδ2 T cells are a major source of interleukin-9. *Proc. Natl. Acad. Sci. U.S.A.* 113 12520–12525. 10.1073/pnas.1607136113 27791087 PMC5098669

[B75] Podbielska M. O’Keeffe J. Hogan E. L. (2018). Autoimmunity in multiple sclerosis: role of sphingolipids, invariant NKT cells and other immune elements in control of inflammation and neurodegeneration. *J. Neurol. Sci.* 385 198–214. 10.1016/j.jns.2017.12.022 29406905

[B76] Rakoff-Nahoum S. Paglino J. Eslami-Varzaneh F. Edberg S. Medzhitov R. (2004). Recognition of commensal microflora by toll-like receptors is required for intestinal homeostasis. *Cell* 118 229–241. 10.1016/j.cell.2004.07.002 15260992

[B77] Ribet D. Cossart P. (2015). How bacterial pathogens colonize their hosts and invade deeper tissues. *Microbes Infect.* 17 173–183. 10.1016/j.micinf.2015.01.004 25637951

[B78] Ristori G. Romano S. Cannoni S. Visconti A. Tinelli E. Mendozzi L. (2014). Effects of Bacille Calmette-Guerin after the first demyelinating event in the CNS. *Neurology* 82 41–48. 10.1212/01.wnl.0000438216.93319.ab 24306002 PMC3873620

[B79] Rumah K. R. Linden J. Fischetti V. A. Vartanian T. (2013). Isolation of *Clostridium perfringens* type B in an individual at first clinical presentation of multiple sclerosis provides clues for environmental triggers of the disease. *PLoS One* 8:e76359. 10.1371/journal.pone.0076359 24146858 PMC3797790

[B80] Rumah K. R. Ma Y. Linden J. R. Oo M. L. Anrather J. Schaeren-Wiemers N. (2015). The myelin and lymphocyte protein MAL is required for binding and activity of *Clostridium perfringens* ε-toxin. *PLoS Pathog.* 11:e1004896. 10.1371/journal.ppat.1004896 25993478 PMC4439126

[B81] Sakai R. E. Feller D. J. Galetta K. M. Galetta S. L. Balcer L. J. (2011). Vision in multiple sclerosis: the story, structure-function correlations, and models for neuroprotection. *J. Neuroophthalmol.* 31 362–373. 10.1097/WNO.0b013e318238937f 22089500 PMC3427931

[B82] Salama N. R. Hartung M. L. Müller A. (2013). Life in the human stomach: persistence strategies of the bacterial pathogen *Helicobacter pylori*. *Nat. Rev. Microbiol.* 11 385–399. 10.1038/nrmicro3016 23652324 PMC3733401

[B83] Schmutzhard E. Pohl P. Stanek G. (1988). *Borrelia burgdorferi* antibodies in patients with relapsing/remitting form and chronic progressive form of multiple sclerosis. *J. Neurol. Neurosurg. Psychiatry* 51 1215–1218. 10.1136/jnnp.51.9.1215 3225603 PMC1033030

[B84] Seno A. Maruhashi T. Kaifu T. Yabe R. Fujikado N. Ma G. (2015). Exacerbation of experimental autoimmune encephalomyelitis in mice deficient for DCIR, an inhibitory C-type lectin receptor. *Exp. Anim.* 64 109–119. 10.1538/expanim.14-0079 26176030 PMC4427725

[B85] Sewell D. L. Reinke E. K. Co D. O. Hogan L. H. Fritz R. B. Sandor M. (2003). Infection with *Mycobacterium bovis* BCG diverts traffic of myelin oligodendroglial glycoprotein autoantigen-specific T cells away from the central nervous system and ameliorates experimental autoimmune encephalomyelitis. *Clin. Diagn. Lab. Immunol.* 10 564–572. 10.1128/CDLI.10.4.564-572.2003 12853387 PMC164279

[B86] Shahi S. K. Freedman S. N. Mangalam A. K. (2017). Gut microbiome in multiple sclerosis: the players involved and the roles they play. *Gut Microbes* 8 607–615. 10.1080/19490976.2017.1349041 28696139 PMC5730390

[B87] Sheu J. J. Lin H. C. (2013). Association between multiple sclerosis and chronic periodontitis: a population-based pilot study. *Eur. J. Neurol.* 20 1053–1059. 10.1111/ene.12103 23398363

[B88] Shimada K. Crother T. R. Arditi M. (2012). Innate immune responses to *Chlamydia pneumoniae* infection: role of TLRs, NLRs, and the inflammasome. *Microbes Infect.* 14 1301–1307. 10.1016/j.micinf.2012.08.004 22985781 PMC3511600

[B89] Sloane J. A. Batt C. Ma Y. Harris Z. M. Trapp B. Vartanian T. (2010). Hyaluronan blocks oligodendrocyte progenitor maturation and remyelination through TLR2. *Proc. Natl. Acad. Sci. U.S.A.* 107 11555–11560. 10.1073/pnas.1006496107 20534434 PMC2895128

[B90] Soos J. M. Schiffenbauer J. Johnson H. M. (1993). Treatment of PL/J mice with the superantigen, staphylococcal enterotoxin B, prevents development of experimental allergic encephalomyelitis. *J. Neuroimmunol.* 43 39–43. 10.1016/0165-5728(93)90073-8 8096223

[B91] Sriram S. Yao S. Y. Stratton C. Moses H. Narayana P. A. Wolinsky J. S. (2005). Pilot study to examine the effect of antibiotic therapy on MRI outcomes in RRMS. *J. Neurol. Sci.* 234 87–91. 10.1016/j.jns.2005.03.042 15935383

[B92] Steed A. L. Stappenbeck T. S. (2014). Role of viruses and bacteria-virus interactions in autoimmunity. *Curr. Opin. Immunol.* 31 102–107. 10.1016/j.coi.2014.10.006 25459001 PMC4254666

[B93] Tang Y. W. Sriram S. Li H. Yao S. Y. Meng S. Mitchell W. M. (2009). Qualitative and quantitative detection of *Chlamydophila pneumoniae* DNA in cerebrospinal fluid from multiple sclerosis patients and controls. *PLoS One* 4:e5200. 10.1371/journal.pone.0005200 19357786 PMC2664471

[B94] Tracy K. E. Baumgarth N. (2017). *Borrelia burgdorferi* manipulates innate and adaptive immunity to establish persistence in rodent reservoir hosts. *Front. Immunol.* 8:116. 10.3389/fimmu.2017.00116 28265270 PMC5316537

[B95] Tremlett H. Fadrosh D. W. Faruqi A. A. Hart J. Roalstad S. Graves J. (2016). Gut microbiota composition and relapse risk in pediatric MS: a pilot study. *J. Neurol. Sci.* 363 153–157. 10.1016/j.jns.2016.02.042 27000242 PMC4806409

[B96] Tremlett H. Waubant E. (2017). The multiple sclerosis microbiome? *Ann. Transl. Med.* 5:53. 10.21037/atm.2017.01.63 28251132 PMC5326653

[B97] Waites K. B. Talkington D. F. (2004). *Mycoplasma pneumoniae* and its role as a human pathogen. *Clin. Microbiol. Rev.* 17 697–728. 10.1128/CMR.17.4.697-728.2004 15489344 PMC523564

[B98] Weir R. E. Gorak-Stolinska P. Floyd S. Lalor M. K. Stenson S. Branson K. (2010). Role of innate immunity in *Helicobacter pylori*-induced gastric malignancy. *Physiol. Rev.* 90 831–858. 10.1152/physrev.00039.2009 20664074 PMC2990353

[B99] Weiss G. Schaible U. E. (2015). Macrophage defense mechanisms against intracellular bacteria. *Immunol. Rev.* 264 182–203. 10.1111/imr.12266 25703560 PMC4368383

[B100] Whiteside S. K. Snook J. P. Ma Y. Sonderegger F. L. Fisher C. Petersen C. (2018). IL-10 deficiency reveals a role for TLR2-dependent bystander activation of t cells in lyme arthritis. *J. Immunol.* 200 1457–1470. 10.4049/jimmunol.1701248 29330323 PMC5809275

[B101] Wucherpfennig K. W. (2001). Mechanisms for the induction of autoimmunity by infectious agents. *J. Clin. Invest.* 108 1097–1104. 10.1172/JCI200114235 11602615 PMC209539

[B102] Yao G. Wang P. Luo X. D. Yu T. M. Harris R. A. Zhang X. M. (2016). Meta-analysis of association between *Helicobacter pylori* infection and multiple sclerosis. *Neurosci. Lett.* 620 1–7. 10.1016/j.neulet.2016.03.037 27033666

[B103] Yoshimura S. Isobe N. Matsushita T. Yonekawa T. Masaki K. Sato S. (2013). Distinct genetic and infectious profiles in Japanese neuromyelitis optica patients according to anti-aquaporin 4 antibody status. *J. Neurol. Neurosurg. Psychiatry* 84 29–34. 10.1136/jnnp-2012-302925 23038741

[B104] Zamvil S. S. Spencer C. M. Baranzini S. E. Cree B. A. C. (2018). The gut microbiome in neuromyelitis optica. *Neurotherapeutics* 15 92–101. 10.1007/s13311-017-0594-z 29280091 PMC5794705

[B105] Zhou Y. Fang L. Peng L. Qiu W. (2017). TLR9 and its signaling pathway in multiple sclerosis. *J. Neurol. Sci.* 373 95–99. 10.1016/j.jns.2016.12.027 28131238

